# Data Dissemination in VANETs Using Particle Swarm Optimization

**DOI:** 10.3390/s23042124

**Published:** 2023-02-13

**Authors:** Dhwani Desai, Hosam El-Ocla, Surbhi Purohit

**Affiliations:** Department of Computer Science, Lakehead University, Thunder Bay, ON P7B 5E1, Canada

**Keywords:** mobile, network, routing, VANET, dissemination, swarm, optimization, ad hoc, ant colony

## Abstract

A vehicular Ad-Hoc Network (VANET) is a type of Mobile Ad-Hoc Networks (MANETs) that uses wireless routers inside each vehicle to act as a node. The need for effective solutions to urban traffic congestion issues has increased recently due to the growing number of automobile connections in the car communications system. To ensure a high level of service and avoid unsafe situations brought on by congestion or a broadcast storm, data dissemination in a VANET network requires an effective approach. Effective multi-objective optimization methods are required to tackle this because of the implied competing nature of multi-metric approaches. A meta-heuristic technique with a high level of solution interactions can handle efficient optimization. To accomplish this, a meta-heuristic search algorithm particle optimization was chosen. In this paper, we have created a network consisting of vehicles as nodes. The aim is to send emergency messages immediately to the stationary nodes. The normal messages will be sent to the FIFO queue. To send these messages to a destination node, multiple routes were found using Time delay-based Multipath Routing (TMR) method, and to find the optimal and secure path Particle Swarm Optimization (PSO) is used. Our method is compared with different optimization methods such as Ant Colony Optimization (ACO), Firefly Optimization (FFO), and Enhanced Flying Ant Colony Optimization (EFACO). Significant improvements in terms of throughput and packet loss ratio, reduced end-to-end delay, rounding overhead ratio, and the energy consumption are revealed by the experimental results.

## 1. Introduction

The vehicular ad hoc network (VANET) is a variant of a mobile ad hoc network (MANET) that uses wireless network technologies to design communication for message flow between adjacent vehicles to vehicles (V2V), vehicles to roadside infrastructures (V2I), and through vehicle-to-vehicle (V2V) and vehicle-to-infrastructure (V2I) connectivity [1]. Vehicular networks have emerged in recent years as a possible data communication option for intelligent transportation systems. The mobility of cars, however, is one of the elements that have a considerable impact on the architecture of these networks. Furthermore, because different vehicles have unique mobility characteristics in urban settings, it becomes much more complicated [2]. In this regard, it is crucial to make use of knowledge of these vehicles’ movement to offer strong solutions and a variety of applications [3].

Accidents and delays in transportation have become inevitable due to the rapidly increasing number of cars, the over-saturation of the transportation infrastructure, and traffic congestion. VANET has been exhibited as a promising solution that can boost a driver’s awareness and provides vehicles with tremendous up-to-date information to prevent accidents and traffic congestion [4]. These systems allow for the construction of several applications that, by utilizing data shared across road agents, empower entities (such as drivers) to make more informed decisions, either manually or automatically [5].

VANET Architecture is made up of highly mobile vehicles called nodes that have sensors called On-Board Units (OBU), which are specialized dedicated sensors. Sensors gather data from stationary Road Side Units (RSU) or other moving vehicles and transmit it directly to other cars or indirectly by passing it to RSUs. Passengers can travel safely and comfortably thanks to VANET. These nodes in VANETs are fitted with sensors that gather data in real-time and transmit it to other moving vehicles or stationary RSUs. The basic structure of a VANET is made up of groups of stationary or moving cars connected via wireless and processing capabilities to create a dynamic vehicular network while traveling on highways. It primarily offers car nodes decentralized data communication. These networks’ primary goals are to support traffic monitoring systems and sustain network effectiveness by enhancing data communication procedures and wireless communication channel performance. The fundamental VANET components, the several forms of communication that can occur between VANET nodes, and the benefits of employing VANET in ITS are all briefly covered in the sections that follow [5].

The main and basic applications of VANETs that enable the enhancement of the efficiency and safety of contemporary transportation systems are those related to safety, commerce, convenience, and productive applications [6,7].

Designing a trustworthy message dissemination protocol that can deliver effective message dissemination, especially for safety-related applications, is a difficult task on the VANET. Network connectivity, which ensures that a message from one car can be forwarded to reach all vehicles in the network, is necessary for the creation of an efficient dissemination protocol [8].

As the VANET network grows, security and privacy problems must be effectively handled. The VANET architecture is susceptible to numerous assaults, including protocol tunneling, eavesdropping, the disclosure of personal information, phony messages that clog up traffic, and the unlawful use of sensitive data [9]. VANETs’ primary goals are to increase road safety, significantly reduce the number of accidents, and boost safety through emergency warnings. These apps unquestionably avert accidents by giving drivers forewarning. Emergency braking, post-crash alerts, real-time traffic, traffic violation warnings, road hazard control alerts, cooperative message transfer, pre-crash sensing, and cooperative collision warning are the main safety applications.

To make the most of the road’s capacity and avoid traffic jams, the driver should be given the information necessary to make decisions and secure the journey. The general goal of convenience applications, such as navigation, active prediction, personal routing, road congestion advice, electronic toll collection, parking availability information, road congestion management, etc., is to manage traffic more conveniently for drivers to increase efficiency. These apps make it possible to prevent traffic issues and maintain a steady flow of traffic.

VANET experts are always attempting to reduce data redundancy to increase the scalability of data dissemination. To overcome scaling issues and create reliable and strong intelligent transportation networks, novel ways of assessing traffic density are required [10]. Despite a plethora of suggestions and the development of communication technology, exact algorithms cannot solve the routing problem in VANETs in polynomial time. Numerous mathematical and computational methodologies have employed optimization as a solution to different engineering problems. A crucial computational strategy in optimization is meta-heuristic optimization. It has the strength and efficiency to find the best solutions since it makes use of the hardware processing capability and incorporates a heuristic search for solutions. The ant bee colony algorithm, the firefly algorithm, particle swarm optimization, harmony search optimization, and heterogeneous computing task scheduling using improved harmony search optimization are a few examples of these heuristic solutions [10].

In this paper, we utilize the Particle Swarm Optimization (PSO) method [11] to optimize the routes returned by the Time delay-based Multipath Routing (TMR) mechanism proposed in [12]. An optimized route is selected for data communication between vehicles in VANETs, particularly in case of emergency incidents. PSO uses the fitness values of the vehicles (particles) to calculate the best route among all of the TMR routes pool. PSO offers better solutions with little parameterization, which is its fundamental advantage. The PSO method is based on velocity rather than only position.

The following are the main contributions of this work:

- To create an effective data dissemination technique for broadcasting in the VANET;
- To create a new appliance for VANET broadcasting that will increase the packet delivery ratio;
- To improve the fitness function and improve the selection of better-optimized solutions in VANET broadcasting by using the Particle Swarm optimization technique.

The rest of the paper is arranged as follows: A survey on a literature review is covered in Section 2. Section 3 explains the proposed protocol. The methodology is shown in Section 4. The performance metrics are explained in Section 5. Section 6 has the experimental results. Lastly, the conclusions are provided in Section 7.

## 2. Literature Review

### 2.1. Particle Swarm Optimization

In [13], authors have explained how clusters arise. Nodes that had been attacked were displayed in the Certificate Revocation List (CRL). Before transmission for secure communication, each node is verified by the Certificate Authority (CA). Following validation, the data are sent from the source node through the best path to the destination node. Utilizing the upgraded OLSR routing protocol, the path was created. PSO is utilized for the best MPR (Multi-point relay) selection. According to the simulation results, the network’s energy efficiency increased when the suggested OLSR-PSO routing technique is used. However, the overhead cost is a challenge in this method where the validation process consumes the network resources in terms of bandwidth and time.

In [14], authors have introduced a two-stage multi-swarm particle swarm optimizer (TMPSO), which makes use of the multi-swarm approach and uses two separate search strategies during all iterations. Unconstrained TMPSO (uTMPSO) and constrained TMPSO (cTMPSO) global optimizations are the two versions of this novel optimizer. The cTMPSO version of the uTMPSO, which replaces the conventional penalty function method, is further improved to handle the constraints by using the trial and error method. Constraint violations are checked on each newly created particle in the aforementioned uTMPSO procedures, and the violating ones are forced to execute "retreat" operations, return to the feasible region, and recreate new positions. However, this method is multi-swarm which requires more processing time than the individual-swarm optimization technique.

The conventional particle swarm optimization process, according to their explanation, will prematurely stagnate as a result of the particle diversity limit and will thereafter reach a local optimum. In [15], authors suggested an optimization approach for particle swarms based on a particle swarm factor selection strategy (FPSO) to address the aforementioned drawbacks. Different parameter selection procedures are used at different phases of the particle swarm search process to enhance the capability of the global search. The outcomes of the simulation demonstrate how effective and better the strategy is at resolving complicated problem optimization. The algorithm’s accuracy over time for high-dimensional test functions stays high. FPSO enhances the average optimization effect; however, it runs longer than the traditional PSO due to the factor selection technique. To enhance the FPSO in terms of the optima convergence, particle population should be enlarged using optimization techniques such as mutation [16].

In [17], the authors propose a large-scale bi-level particle swarm optimization algorithm that expands the particle swarm scale and improves the initial population diversity based on multi-particle swarms. This algorithm is targeted at the slow convergence and the local optimum problems of PSO. A bi-level particle swarm’s structural benefits, on the other hand, allow this method to increase the operating efficiency of particle swarms by allowing the upper-level particle swarm to give decision-making information while the lower-level working particle swarms operate concurrently. The large-scale bi-level particle swarm optimization approach has been shown through simulated trials to provide satisfactory optimization results. However, the stability of the algorithm keeps changing in terms of data throughput.

In [18], an easy particle was suggested, which is motivated by the effect of a lazy ant in the ant colony for PSO to address the constraint issue in Nonlinear Constrained Optimization (NCO) problems. The easy particle is very convenient to embed in the current PSO-based techniques. The proposed easy particle, which is unrestrained by social and cognitive characteristics and exhibits lazy ant behavior, can overcome constraint containment to improve the possibility of exploring uncharted territory. The experiments demonstrate that the suggested simple particles integrated into the referred algorithms can successfully reduce premature convergence, thereby greatly enhancing NCO problem performance. Refreshing the number of simple particles as needed sounds like a wise course of action.

The PSOR routing protocol is proposed in [19], and it uses the vehicle’s distance and speed to locate the next forwarding vehicle. PSOR is proven to perform efficiently, particularly when compared with AntHocNet and Adaptive QoS-based Routing for VANETs (AQRV). However, they are not considering highway scenarios where vehicle nodes have high speeds.

The study in [20] focuses on using an efficient system to distribute tasks among vehicles based on position and velocity. With PSO, each vehicle is used more efficiently, resulting in higher average resource utilization. However, this method did not consider the fair load distribution among the vehicles so some are overloaded while others would stay idle.

The algorithm presented in [21,22] is a hybrid metaheuristic optimization algorithm that combines the Particle Filter (PF) and Particle Swarm Optimization (PSO) algorithms. The new Particle Filter-Particle Swarm Optimization (PF-PSO) algorithm is applied to the optimal tuning of Proportional-Integral-fuzzy controllers for the position control of a family of integral-type servo systems. However, energy consumption is augmented due to the fuzzy control system operation, and this accordingly reduces the lifespan of the vehicle nodes.

### 2.2. Data Dissemination

**Figure 1:**
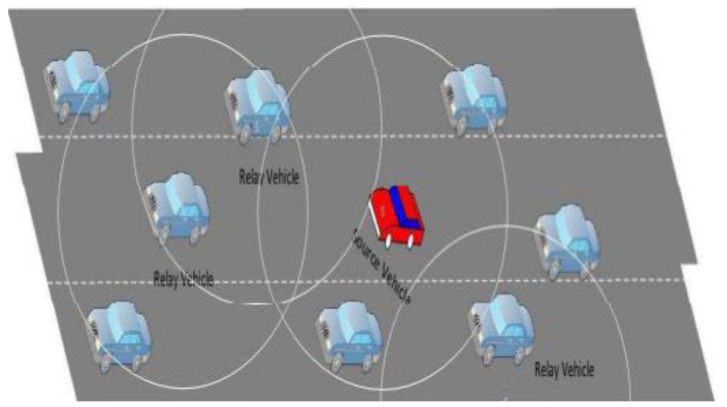
Data Dissemination in VANET [22].

The virtual representation of Data dissemination in the Vehicular Ad-Hoc network is displayed in Figure 1.

In [23], it is explored how to best choose which vehicle to inject data into, when to do so, and whether to have the vehicle collect the required data directly from the edge or adjacent neighbors to reduce edge traffic costs and satisfy data acquisition deadlines. The current methodology gives priority to choosing V2I dissemination first before exploring V2V dissemination that does not clash with V2I dissemination. This method falls short of fully utilizing V2V to lower edge traffic costs. The authors have proposed a new data dissemination algorithm, called the offline algorithm for hybrid data dissemination (OFDD), which seeks the most beneficial V2V broadcasts with priority, and then chooses feasible V2I dissemination. Based on OFDD, both the snapshot and prediction-based online algorithms were developed. However, the performance of the vehicle densities and data request load is affected by this method.

In [24], the authors proposed an Efficient multi-directional Data Dissemination Protocol (EDDP), which considers the requirements of an urban vehicular environment without requiring the extra communication overhead. Simple local data only were used to indicate the road condition for better dissemination performance. In their proposal, the design is considering urban layout including message format, broadcast suppression mechanism, and delay control. The EDDP utilizes the properties of the received messages along with positioning information to make decisions on suppressing broadcasts, and to improve coverage in different directions without unnecessary transmissions. EDDP can effectively disseminate traffic data with a high data delivery ratio and a minimized overhead. However, and in terms of coverage, data redundancy, and dissemination latency, EDDP did not address the broadcast storm problem with various layout considerations.

In [25], the authors have proposed a novel unmanned aerial vehicle (UAV)-enabled scheduling protocol consisting of a proactive caching policy and a file-sharing strategy in V2X networks to empower the efficiency of data dissemination. In the proactive caching process, it was suggested to deploy UAVs as flying base stations (BSs) with caching capability. A UAV dynamic trajectory scheduling (DTS) algorithm was introduced to optimize the caching duration. However, in the file sharing strategy, based on the previous vehicular caching status, this model provides a framework of file sharing cycle for data dissemination scheduling and employs a channel prediction algorithm to alleviate communication overhead. Moreover, this method proposes a relay ordering algorithm to effectively improve the file-sharing process. However, with increasing the number of vehicles, the throughput is reduced obviously, and this can lead to low stability for dissemination problems.

In [26], the authors considered a cognitive unmanned aerial vehicle (UAV) to disseminate data to a group of Internet of Things (IoT) devices. The cognitive UAV shares the wireless spectrums of primary users (PUs) where it accesses the spectrums opportunistically when the corresponding channels are available. The UAV predicts the OFF periods of the PU; then, it decides the number of consecutive transmission slots to be used for data transmission. To protect the PUs from harmful interference, the number of slots used for transmission should not exceed a certain threshold. The formulated problem is in a form of a mixed integer nonlinear program (MINLP). Therefore, a successive convex approximation-based algorithm was proposed to solve this problem after approximating it to a convex problem. However, topology change would degrade the network performance.

In [27], the authors introduced a novel approach known as Named Data Networking (NDN). It enables scalable, efficient, and secure data distribution for a variety of applications. NDN is a data-centric communication protocol that combines content naming, name-based routing and forwarding, in-network caching, and data-centric security. It is based on simple and effective working principles. This method aims to present a multi-layered framework as a multiple-phase systematic approach for designing an efficient VANET-NDN data dissemination scheme. However, the network performance in terms of throughput and latency is not that high.

In [28], a Travel Angle-based Fast Data Dissemination to Relevant Vehicles (TAFDRV) protocol was proposed to control the direction of data dissemination. The design goal of this protocol is to distribute information among relevant vehicles. However, the information may not be equally relevant for all vehicles in the scenario, and this will lead to network congestion.

In [29], it was proposed a MultiCriteria-based Relay Election Protocol for Data Dissemination in Urban VANETs (MCRE-DDP). The most relevant relay node is chosen based on several parameters such as Signal Noise Ratio (SNR), vehicle speed, and distance between sender and receiver to determine the node’s quality and ability to successfully relay the dissemination message. Relevant relays prevent the communication system from multiple sending and guarantee the appropriate message delivery. However, the rounding overhead ratio is comparatively high as more packets are required for network maintenance.

In [30], for efficiently distributing deadline-sensitive streaming files in VANETs, a mechanism (dubbed TDDV) for disseminating timely P2P segments is proposed. This mechanism enhances the QoS performance; however, the high mobility speed of nodes is not tolerated.

## 3. Proposed Protocol

### 3.1. Problem Statement

The data dissemination is a promising application for VANETs. Most existing data dissemination strategies typically rely on a random-access protocol, which leads to a collision problem that cannot be avoided. Another issue is the risk of delay, which is not meeting the real-time restrictions on managing an emergency. Hence, the logic of data dissemination must be accurate and checks if all the requirements of efficient delivery are met. This includes reducing data collisions, preventing bottlenecks, and minimizing the overhead cost, and this should be made possible under time limitations. Data dissemination requires the assurance of optimal routes for delivering messages to their destinations within a certain time interval. The most difficult components of this challenge are assumed to be the different features of optimization in data dissemination, its relationship to the dynamics of message generation and vehicle mobility, as well as the fact of topological change. Thus, to solve this problem, the integration of different optimized models is required.

### 3.2. Proposed Solution

To address the problem of data dissemination in VANETs, we introduce an optimization technique based on PSO while using the TMR routing technique [12]. Instead of selecting the road with the fewest hops, TMR selects the path with the lowest RTT. The second shortest RTT path is utilized to transfer data messages if the first one fails. When evaluating a potential route, the average TMR establishes a threshold value that restricts a measured instant RTT. When RTT or node speed is high, this average RTT is set to prevent overburdening the network with data packets. This protocol is designed to differentiate the normal and emergency messages. Emergency messages are delivered without delay, whereas normal messages are queued and delivered using the TMR protocol. We propose to optimize TMR routes using PSO to transfer the message from the source to the destination with a low delivery delay time because the velocity and the position are considered. In PSO, particles (vehicles) can converge quickly to the best positions. PSO has a fast convergence speed in finding the global optimum for uni-modal and simple multi-modal problems. Particles are easily trapped into local minima when solving complex multi-modal problems. As a result, all particles will rapidly converge to the local optimum. Finally, premature convergence takes place. This paper embeds neighborhood search strategies in standard PSO to improve its performance in solving complex problems.

### 3.3. Network Configuration

**Figure 2:**
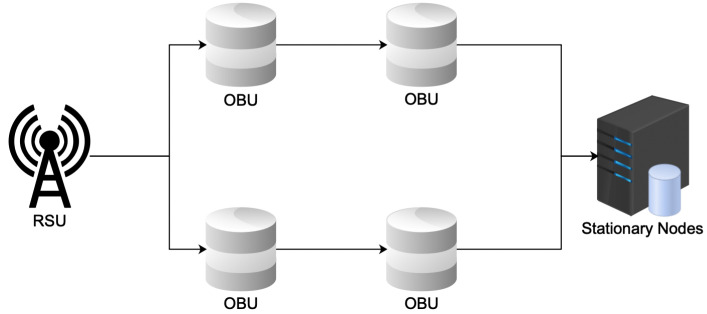
Network configuration.

In the scenario depicted in Figure 2, we considered a situation in which data communication occurs between OBUs and the RSU close to it in one road segment [31]. The study’s objective in this scenario is to evaluate the effectiveness of each protocol within a road segment. The stationary nodes, such as hospitals and fire stations, are the destination node for the emergency message transfer.

## 4. Methodology

In this paper, the PSO technique is used for fast searching and optimization. For a better understanding of our proposed protocol, we first explain to the network model as follows: The series of vehicles in a network is denoted as $X=x_{1},x_{2},\cdots ,x_{n}$, where *n* is the maximum number of vehicles in the network. The vehicles’ velocity is denoted as $V=v_{1},v_{2},\cdots ,v_{n}$. The position of the vehicles is referred as $D_{i}=d_{i1},d_{i2},\cdots ,d_{in}$. To find the best solutions, the PSO algorithm [32] uses velocity to change (evolution) a particle’s position. The most frequent way to control particle velocity is to multiply it by a factor. *A* is the array of routes returned by TMR. Every particle has a unique velocity and position, which are first established at random. The pbest, or local best position, and gbest, or global best position, of every particle, must be maintained.

**Figure 3:**
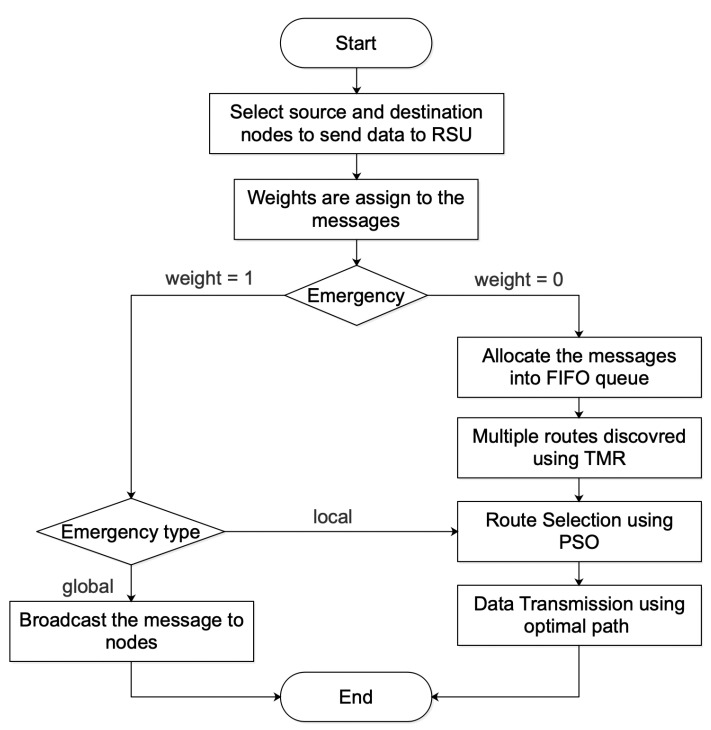
Flow diagram of the network.

The virtual representation of the flowchart is given in Figure 3:

- We start with *n* vehicle nodes, some RSUs and OBUs connected in the same network. The message forwarding process between RSU and OBUs occurs when the vehicles are moving in the same area zone.
- These messages are divided into two parts which can be distinguished by their weights. If the weight = 1, then it is the emergency message which needs immediate assistance. Source or destination ends may be mobile nodes which are referred to as OBUs. On the other hand, the messages with weight = 0 will directly transfer to the FIFO queue for further data transmission. For these message transmissions, multiple routes were found using TMR [12].
- Vehicles send both routine and urgent communications to RSUs, which then transmit them to other vehicles [33]. In the case of unicast communication, TMR, which is a multipath routing technique based on Round Trip Time (RTT), is utilized, and it is primarily concerned with cutting down the end-to-end time delay.
- We assume that the initial position and velocity can be determined using the following equations:
  Each node that joins the network should announce its $v_{i}$. PSO is applied on routes returned by TMR. For each TMR’s route, node *i* will send hello messages to its neighbor nodes *j* recorded in the TMR routes. $T_{ij}$ is the time between one node and its neighbor node.
  Therefore, the Euclidean distance between nodes *i* and *j*, for a given route, can be calculated as:
  (1) $$d_{ij}=v_{j}\times T_{ij}$$
  where $v_{j}$ is the velocity of the neighbor vehicle *j* compared to previous node *i*. Alternatively, inter-distance between nodes can be calculated as in [34]. Hence, the best position (pbest) between one node and its adjacent *l* node in a given route out of *k* TMR routes is:
  (2) $$pbest=\underset{l}{\mathrm{Max}}\left(d_{ij}^{l}\right)$$
  The pbest is calculated with the maximum distance between the source node and the nodes in a given TMR route to consider the closest node to the destination vehicle/RSU.
  From one iteration to the next, a swarm of particles (nodes) updates their relative positions, giving the PSO algorithm the boost it needs to properly search. Each particle advances toward its previous personal best position (pbest) to achieve the best result.
- The optimization function is defined in terms of fitness function (*FF*). This FF is computed to determine the next forwarding vehicle that uses parameters such as the position of the vehicle, its velocity, and distance to its adjacent vehicle. The source node finds the adjacent node closer to the destination, and its fitness value is determined. The adjacent vehicle having the highest fitness value is selected as the next forwarding node and the packet is forwarded to it. The process is repeated iteratively for all TMR routes until the packet reaches the destination node [35]. The fitness function is calculated for each vehicle according to its position and velocity. *FF* is represented in Equation (3) given below [36]:
  (3) $$FF=\sum_{j=1}^{l}w_{1}\cdot d_{ij}+w_{2}\cdot v_{i}$$
  where $w_{1},w_{2}$ are the weights, *l* is the number of all nodes in each TMR’s route, and i,j are two adjacent nodes.

Each particle is considered as a vehicle where its position *d* and velocity *v* are updated using Equations (4) and (5), respectively:

(4) d(t+1)=d(t)+v(t+1)

(5) $$v\left(t+1\right)=w\cdot v\left(t\right)+\left(c_{1}\cdot r_{1}\cdot pbest\right)+\left(c_{2}\cdot r_{2}\cdot gbest\right)$$

where *t* stands for the iteration index representing each TMR’s route. In other words, d(t+1) is the updated position of the previous position at d(t) and similarly for velocity v(t+1). *w*, $c_{1}$, $c_{2}$ are acceleration coefficients. Acceleration coefficients are critical parameters of the PSO algorithm, which is used to control vehicles’ movement by modifying its cognitive and social components. $r_{1}$, $r_{2}$ are random uniform numbers in between 0 and 1, pbest is at the previous position d(t) of the vehicle (local best) and gbest is the pbest at the updated position d(t+1) of vehicle (global best) [37].

Algorithm 1 shows our mechanism which is explained as follows:

1. The routes were given using TMR as input to look for the best solution.
2. Every vehicle has its distinct position (quality of solution) and velocity.
3. Each vehicle selects the best position and uses the velocity of the vehicle to move towards a new best position. The adjustment of the position to the global optimum is made feasible using the best position of individuals and the whole swarm.
4. At each iteration, each vehicle updates its velocity and position.
5. For each vehicle, the fitness value of each vehicle is computed and compared with the fitness value in the memory. If the current fitness value is greater, then it acts as a global optimum.
6. The procedure stops after the last iteration. The best solution is considered the global best.
7. A vehicle with the maximum global optimum is the next forwarding node and the packet is forwarded towards it.
8. The process is repeated till the packet reaches the destination.

**Algorithm 1:** Message forwarding process.**Initialization:**Array of *k* routes by TMR is A[]Best position pbestCurrent position & velocity of vehicle $d_{ij}\left(t\right)$ & $v_{i}\left(t\right)$Next, position & velocity of vehicle $d_{ij}\left(t+1\right)$ & $v_{i}\left(t+1\right)$1:Initialize pbest2:k=13:**while***A***do**4:   Evaluate FF as Equation (3) for *l* vehicles in *k* route5:   **if** $pbest<d_{ij}\left(t+1\right)$ **then**6:     pbest = $d_{ij}\left(t+1\right)$7:   **end if**8:   Update $d_{ij}\left(t+1\right)$ with Equation (4)9:   Update $v_{i}\left(t+1\right)$ with Equation (5)10:   k=k+111:**end while**

## 5. Performance Evaluation

### 5.1. Simulation Parameters

The parameters and their values are shown in Table 1.

### 5.2. Performance Metrics

#### 5.2.1. Throughput

Throughput of the VANET has successfully received packets in terms of Mbps. Mathematically throughput can be represented as [38]:

(6) $$T=\frac{S_{P}}{T_{L}}\times 10^{-6}\left(\mathrm{Mbps}\right)$$

where *T* represents the throughput, $S_{P}$ is the number of successfully transmitted packets, and $T_{L}$ is simulation time.

#### 5.2.2. Rounding Overhead

It denotes the number of routing packets that must be broadcasted for route discovery and route maintenance to deliver data packets [39]. It can be represented as:

(7) $$RO=\frac{R_{P}}{R_{P}+D_{P}}\times 100$$

where *RO* represents routing overhead, $R_{p}$ represents the number of routing packets, and $D_{p}$ represents the number of data packets sent.

#### 5.2.3. Packet Delivery Ratio

It is calculated by dividing the number of successfully delivered data packets by the total number of data packets generated [39]. It can be expressed mathematically as:

(8) $$PDR=\frac{PR}{PG}\times 100\%$$

where *PDR* represents Packet Delivery Ratio, *PR* is the total data packets received successfully, and *PG* is the total data packets generated.

#### 5.2.4. End to End Delay

The total time spent by the network in a memory buffer, waiting in a queue, packet retransmission, and packet propagation is referred to as End to End Delay. In other words, delay can be represented as the difference in time stamps between packet arrival and transmission [40]. Delay can be represented mathematically as:

(9) $$E2E=\frac{PD}{n}$$

where E2E represents the End-to-End Delay, *PD* is the total time taken by the packets to be successfully delivered since sent out, and *n* is the total number of packets received.

#### 5.2.5. Energy Consumption

The amount of energy consumed by each vehicle node during packet transmission is referred to as energy consumption. It is the difference between the vehicle node’s initial and current energy [40]:

(10) EC=InitialEnergy−CurrentEnergy,

where *EC* represents Energy Consumption.

#### 5.2.6. Gain/Savings

To understand better about gain and savings of our proposed protocol PSO compared with other protocols which are ACO, FFO, and EFACO is calculated using the below equation [41]:

(11) $$Gain/Savings=\pm \left(\left(\sum x_{1i}-\sum x_{2i}\right)/\sum x_{2i}\right)\times 100\left[\%\right]$$

where $x_{1i}$ is the performance value of our protocol; $x_{2i}$ is the performance value of other protocols; ∑ is the summation over the range from *i* = 1 to the number of *x*-axis points of the metric y.

## 6. Experimental Results

Here, we compare our proposed method PSO method with three other methods which are Firefly Optimization (FFO) [42], Ant Colony Optimization (ACO) [43], and Enhanced Flying Ant Colony Optimization (EFACO) [44] in a VANET network.

### 6.1. Throughput

**Figure 4:**
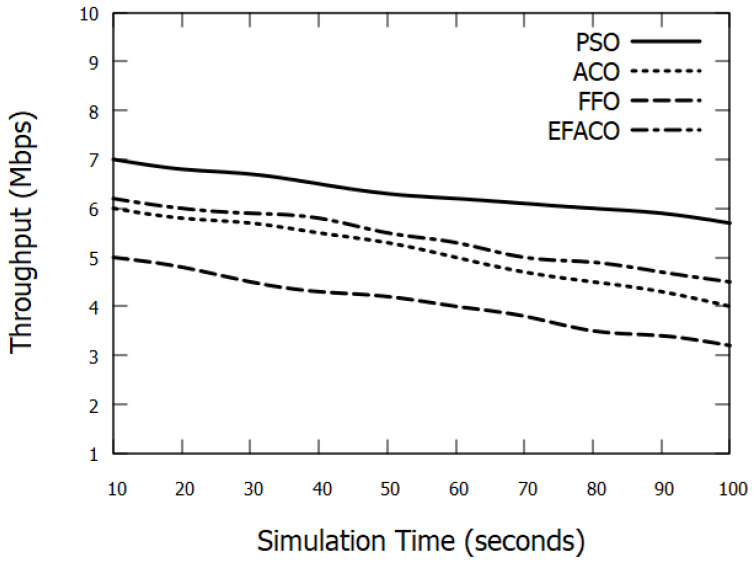
Comparison between proposed PSO, ACO, FFO, EFACO for throughput with simulation time.

Figure 4 depicts the effect of varying simulation time on the throughput. As the simulation time progresses, the data traffic enlarges in the network. Therefore, the packet loss probability increases which in turn reduces the throughput for all protocols. However, our protocol has a higher performance than other protocols.

**Figure 5:**
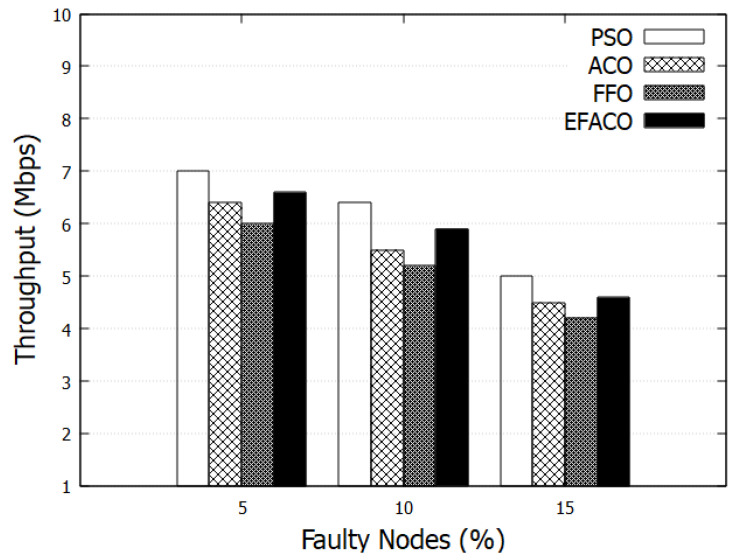
Comparison between proposed PSO, ACO, FFO, EFACO for throughput with faulty nodes.

Figure 5 presents results with the assumption of having faulty nodes in the selected route. When the faulty nodes are present, it becomes harder for the algorithms to progress with the same flow. More packets will be dropped when the number of faulty nodes increases and that, in turn, would degrade the throughput. Here, we have taken three scenarios as 5%, 10%, 15% faulty nodes out of 100 nodes. From the results, it is clear that PSO performs better in comparison to other optimization methods. In addition, we can see that the EFACO method is better than ACO.

**Figure 6:**
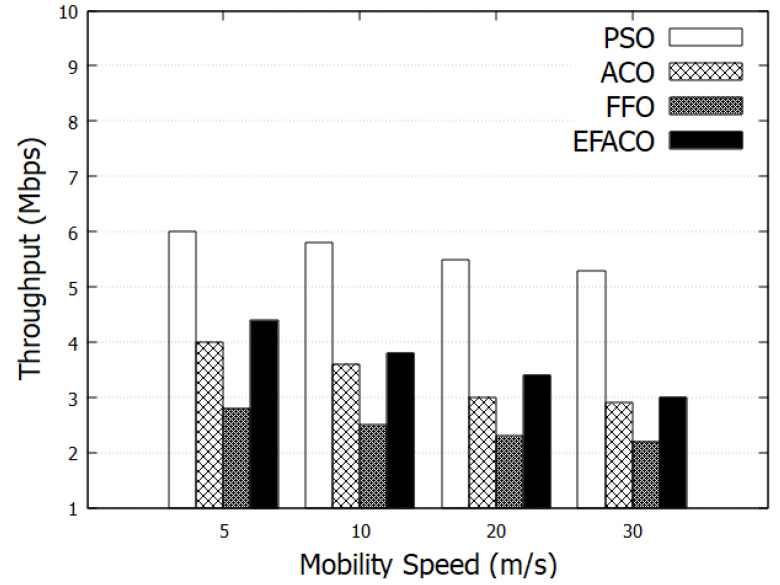
Comparison between proposed PSO, ACO, FFO, EFACO for throughput with mobility speed.

Figure 6 shows the throughput with various mobility speeds of nodes. Nodes having high speeds would result in topological change and packet drops. Accordingly, the throughput degrades with the mobility speed. Our protocol considers the node’s speeds where data will be forwarded to those nodes having the close speed to the forwarding node. As a result, our protocol outperforms ACO, FFO, and EFACO with 67.4%, 130.61%, and 54.97%, respectively, as shown in Table 2.

The number of nodes is assumed 100 as indicated in Table 1. This in turn increases the population and hence the optima convergence is reached with a weak stagnation possibility. Accordingly, the throughput is stable as shown in the above figures.

### 6.2. End to End Delay

**Figure 7:**
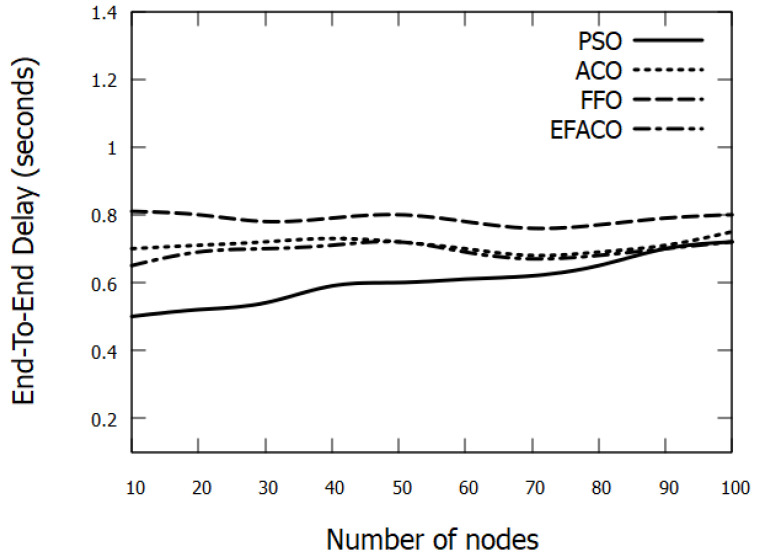
Comparison between proposed PSO, ACO, FFO, EFACO for End-to-End Delay with a number of nodes.

**Figure 8:**
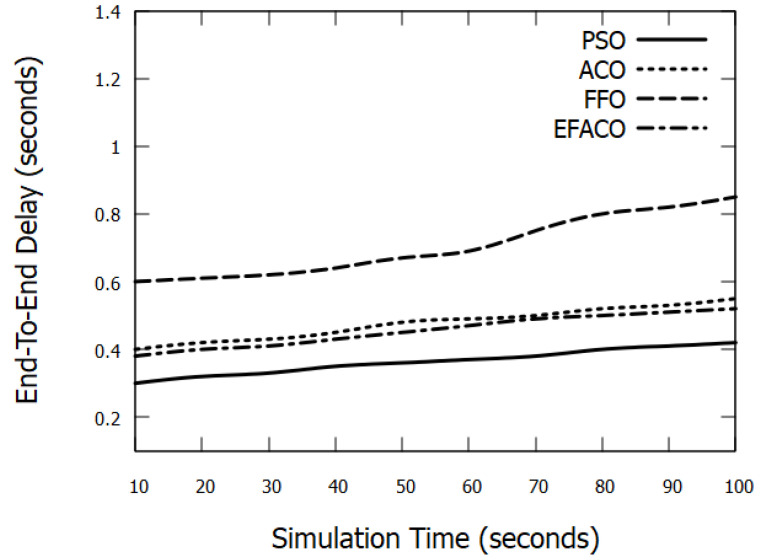
Comparison between proposed PSO, ACO, FFO, EFACO for End-to-End Delay with simulation time.

Results in Figure 7 and Figure 8 represent the performance of End-to-End delay comparing other optimization methods. It is clear that our proposed method PSO is giving better performance because it uses a reliable route so the amount of successfully delivered data *n* in Equation (9) is augmented and accordingly end-to-end delay is reduced. PSO reduces the delay with 15.03%, 23.22%, and 12.69%, compared to ACO, FFO, and EFACO, respectively, as shown in Table 3.

**Figure 9:**
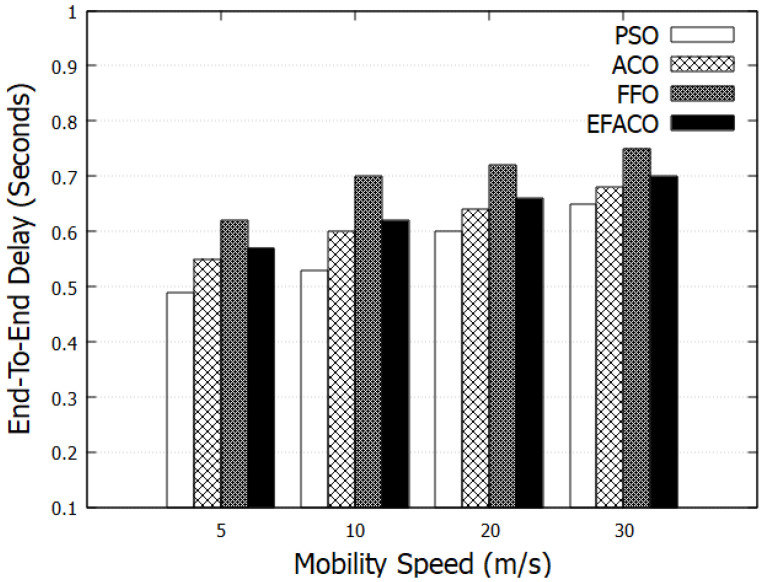
Comparison between proposed PSO, ACO, FFO, EFACO for End-to-End Delay with mobility speed.

**Figure 10:**
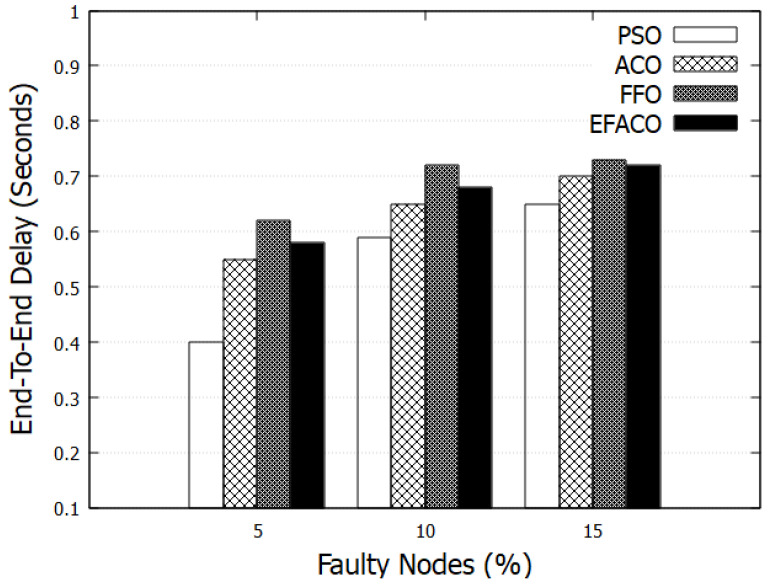
Comparison between proposed PSO, ACO, FFO, EFACO for End-to-End Delay with faulty nodes.

In case of having faulty nodes or vehicles with high speeds, topological change occurs frequently and this in turn reduces the performance of the network due to a higher chance of link failure. Some of the packets are lost and, therefore, retransmissions will augment the end-to-end delay as shown in Figure 9 and Figure 10.

### 6.3. Packet Delivery Ratio

**Figure 11:**
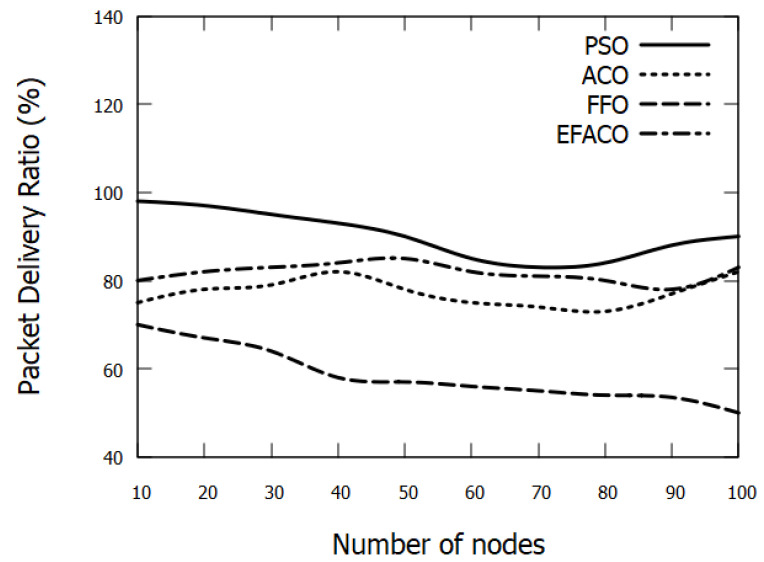
Comparison between proposed PSO, ACO, FFO, EFACO for Packet Delivery Ratio with a number of nodes.

**Figure 12:**
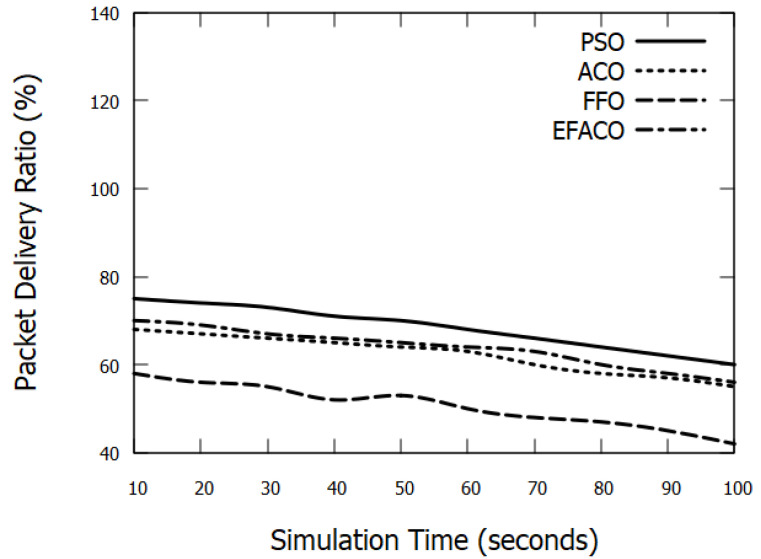
Comparison between proposed PSO, ACO, FFO, EFACO for Packet Delivery Ratio with simulation time.

As the simulation time progresses or the density of nodes augments, more data packets overwhelm the network, and this in turn would result in data loss and more data retransmissions triggered. Accordingly, the performance degrades as shown in Figure 11 and Figure 12. Our mechanism considers the least node’s velocity and the closest nodes to the destination, and this as a result would minimize the possibility of packet drops. PSO enhances the PDR with 16.81%, 54.49%, and 10.39%, compared to ACO, FFO, and EFACO, respectively, as shown in Table 4.

### 6.4. Rounding Overhead

**Figure 13:**
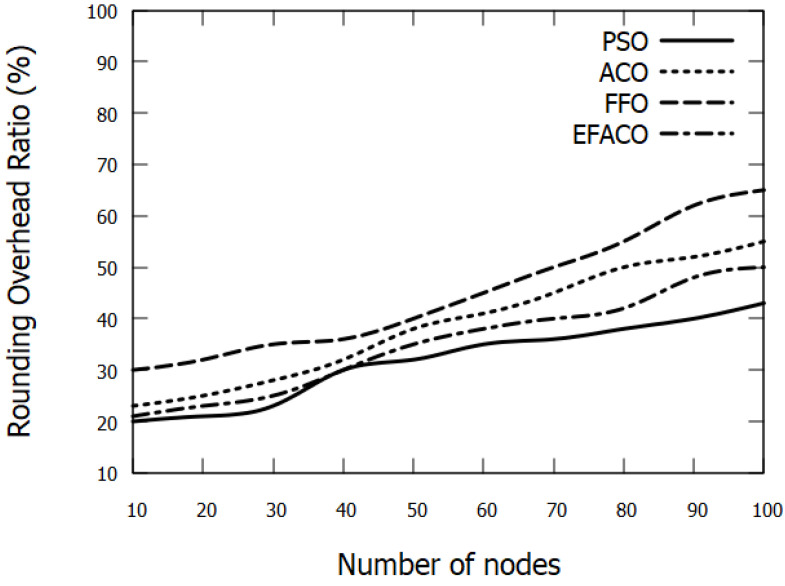
Comparison between proposed PSO, ACO, FFO, EFACO for Rounding Overhead with number of nodes.

**Figure 14:**
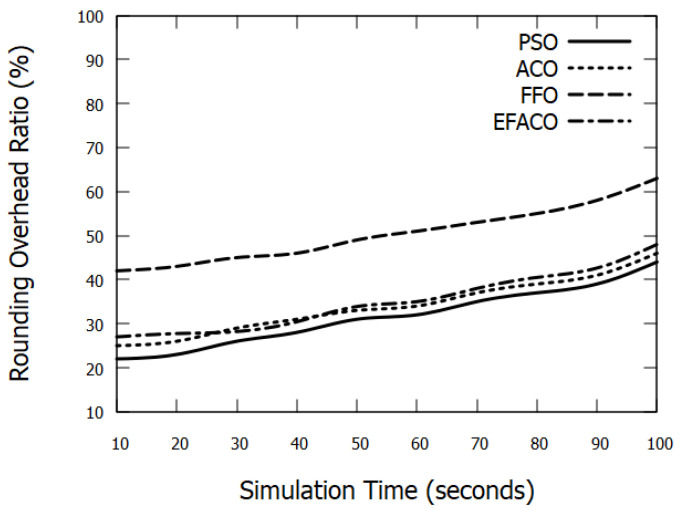
Comparison between proposed PSO, ACO, FFO, EFACO for Rounding Overhead with simulation time.

As the vehicle density increases or simulation time progresses, more data packets are transferred through the network. This as a result would lead to more data drops. Accordingly, the need for new routes will augment requiring an excessive amount of control messages (RREQ/RREP) to seek new routes and this in turn increases the routing overhead as shown in Figure 13 and Figure 14.

### 6.5. Energy Consumption

**Figure 15:**
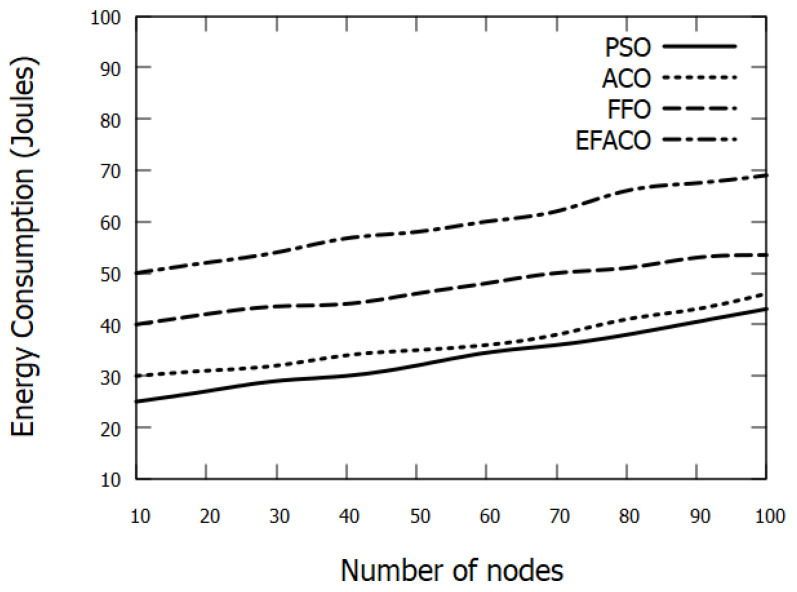
Comparison between proposed PSO, ACO, FFO, EFACO for Energy Consumption with a number of nodes.

When the number of vehicles increases, more packets travel through the network as indicated earlier. As a result, the possibility of data loss augments and retransmissions are triggered, and this will consume more energy. Our mechanism selects the most reliable route in which it will reduce the retransmission rate and this lessens the energy consumption as shown in Figure 15. PSO saves the energy with 8.46%, 28.87%, and 43.72%, compared to ACO, FFO, and EFACO, respectively, as shown in Table 5.

## 7. Conclusions

Vehicular Ad hoc Networks (VANETs) have a wide range of applications requiring secure and efficient data dissemination. Because of the time-varying topology and frequent path disconnections of vehicular communication, a mechanism to provide a reliable path for data transmission from source to destination is probed. In this paper, we propose a routing mechanism that utilizes the time delay-based multipath routing (TMR) method. We apply the particle swarm optimization technique on the routes returned by TMR to select the best reliable path. This method provides stable routes to disseminate data packets in VANETs. Our proposed technique shows great performance in terms of QoS parameters compared to recent routing protocols.

## Figures and Tables

**Table 1:** Simulation parameters.

Parameter Type	Parameter Value
Environment	ns3
Operating System	Ubuntu 14.04
Optimization	Particle Swarm Optimization
Road Type	Highway
Parameters	Throughput, End-to-End Delay, Packet Delivery Ratio, Rounding Overhead, Energy Consumption
Mobility Model	Random Way Point Model
Number of vehicles	100
Mobility Speed	10 m/s
Simulation Time	100 s
Wireless protocol	IEEE 802.11p
Number of faulty nodes	0%
Packet loss ratio	0%
Initial Energy	100J
Traffic type	Constant Bit Rate (CBR)
Comparison Protocols	Firefly Optimization, Ant Colony Optimization, Enhanced Flying Ant Colony Optimization

**Table 2:** Throughput vs. mobility speed in Figure 6.

No. of Nodes	PSO	ACO	FFO	EFACO
5	6	4	2.8	4.4
10	5.8	3.6	2.5	3.8
20	5.5	3	2.3	3.4
30	5.3	2.9	2.2	3
Sum	22.6	13.5	9.8	14.6
Gain %		67.4	130.61	54.97

**Table 3:** End-To-End-Delay in (Seconds) in Figure 7.

No. of Nodes	PSO	ACO	FFO	EFACO
10	0.5	0.7	0.81	0.65
20	0.52	0.71	0.8	0.69
30	0.54	0.72	0.78	0.7
40	0.59	0.73	0.79	0.71
50	0.6	0.72	0.8	0.72
60	0.61	0.7	0.78	0.69
70	0.62	0.68	0.76	0.67
80	0.65	0.69	0.77	0.68
90	0.7	0.71	0.79	0.7
100	0.72	0.75	0.8	0.72
Sum	6.05	7.11	7.88	6.93
Saving %		15.03	23.22	12.69

**Table 4:** Packet Delivery Ratio in (%) in Figure 11.

No. of Nodes	PSO	ACO	FFO	EFACO
10	98	75	70	80
20	97	78	67	82
30	95	79	64	83
40	93	82	58	84
50	90	78	57	85
60	85	75	56	82
70	83	74	55	81
80	84	73	54	80
90	88	77	53.5	78
100	90	82	50	83
Sum	903	773	584.5	818
Gain %		16.81	54.49	10.39

**Table 5:** Energy Consumption in (Joules) in Figure 15.

No. of Nodes	PSO	ACO	FFO	EFACO
10	25	30	40	50
20	27	31	42	52
30	29	32	43.5	54
40	30	34	44	56.75
50	32	35	46	58
60	34.5	36	48	60
70	36	38	50	62
80	38	41	51	66
90	40.5	43	53	67.5
100	43	46	53.5	69
Sum	335	366	471	595.25
Saving %		8.46	28.87	43.72

## Data Availability

Not applicable.

## References

[1] Sharan B., Chhabra M., Sagar A.K. State-of-the-art: Data Dissemination Techniques in Vehicular Ad-hoc Networks. Proceedings of the 2022 9th International Conference on Computing for Sustainable Global Development (INDIACom).

[2] Mohammed S.J., Hasson S.T. Modeling and Simulation of Data Dissemination in VANET Based on a Clustering Approach. Proceedings of the 2022 International Conference on Computer Science and Software Engineering (CSASE).

[3] Celes C., Boukerche A., Loureiro A.A.F. On the Design of Bus-Based Vehicular Networks: Mobility Generation and Data Dissemination. Proceedings of the ICC 2022—IEEE International Conference on Communications.

[4] Hajjej A., Najjar L., Ayaida M., Messai N., Najeh S. Improved Contention Based Forwarding for data broadcasting in VANETs. Proceedings of the 2022 International Wireless Communications and Mobile Computing (IWCMC).

[5] Ribeiro B., Nicolau M.J., Santos A. Leveraging Vehicular Communications in Automatic VRUs Accidents Detection. Proceedings of the 2022 Thirteenth International Conference on Ubiquitous and Future Networks (ICUFN).

[6] RadhaKrishna Karne D.T. (2021). Review on vanet architecture and applications. Turk. J. Comput. Math. Educ..

[7] Noussaiba M., Rahal R. State of the art: VANETs applications and their RFID-based systems. Proceedings of the 2017 4th International Conference on Control, Decision and Information Technologies (CoDIT).

[8] Salem F. Connectivity-Based Dissemination Protocol for VANET. Proceedings of the 2022 IEEE 2nd International Maghreb Meeting of the Conference on Sciences and Techniques of Automatic Control and Computer Engineering (MI-STA).

[9] Hamdi M.M., Audah L., Rashid S.A. (2022). Data Dissemination in VANETs Using Clustering and Probabilistic Forwarding Based on Adaptive Jumping Multi-Objective Firefly Optimization. IEEE Access.

[10] Hajlaoui R., Alaya B., Mchergui A. Optimized VANET Routing Protocol Using Cuckoo Search Algorithm. Proceedings of the 2022 International Wireless Communications and Mobile Computing (IWCMC).

[11] Zhang Q., Liu W., Meng X., Yang B., Vasilakos A.V. (2017). Vector coevolving particle swarm optimization algorithm. Inf. Sci..

[12] Ghaemi Y., El-Ocla H., Yadav N.R., Madana M.R., Raju D.K., Dhanabal V., Sheshadri V. (2021). Intelligent Transport System Using Time Delay-Based Multipath Routing Protocol for Vehicular Ad Hoc Networks. Sensors.

[13] BrijilalRuban C., Paramasivan B. (2021). Energy Efficient Enhanced OLSR Routing Protocol Using Particle Swarm Optimization with Certificate Revocation Scheme for VANET. Wirel. Pers. Commun..

[14] Zhao Q., Li C. (2020). Two-Stage Multi-Swarm Particle Swarm Optimizer for Unconstrained and Constrained Global Optimization. IEEE Access.

[15] Lv J., Shi X. Particle Swarm Optimization Algorithm Based on Factor Selection Strategy. Proceedings of the 2019 IEEE 4th Advanced Information Technology, Electronic and Automation Control Conference (IAEAC).

[16] Stacey A., Jancic M., Grundy I. Particle swarm optimization with mutation. Proceedings of the 2003 Congress on Evolutionary Computation.

[17] Jiang J.-J., Wei W.-X., Shao W.-L., Liang Y.-F., Qu Y.-Y. (2021). Research on Large-Scale Bi-Level Particle Swarm Optimization Algorithm. IEEE Access.

[18] Tseng H.-Y., Chu P.-H., Lu H.-C., Tsai M.-J. (2021). Easy Particle Swarm Optimization for Nonlinear Constrained Optimization Problems. IEEE Access.

[19] Yelure B., Sonavane S. Particle Swarm Optimization based Routing Method for Vehicular Ad-hoc Network. Proceedings of the 2020 International Conference on Communication and Signal Processing (ICCSP).

[20] Keshari N., Gupta T.S., Singh D. Particle Swarm Optimization based Task Offloading in Vehicular Edge Computing. Proceedings of the 2021 IEEE 18th India Council International Conference (INDICON).

[21] Pozna C., Precup R.E., Horváth E., Petriu E.M. (2022). Hybrid Particle Filter-Particle Swarm Optimization Algorithm and Application to Fuzzy Controlled Servo Systems. IEEE Trans. Fuzzy Syst..

[22] Mahmood T. (2021). Data Dissemination Scheme for VANET using Genetic algorithm and Particle Swarm Optimization. Int. J. Recent Technol. Eng..

[23] Yang L., Zhang L., He Z., Cao J., Wu W. (2020). Efficient Hybrid Data Dissemination for Edge-Assisted Automated Driving. IEEE Internet Things J..

[24] Chaqfeh M., El-Sayed H., Lakas A. (2019). Efficient Data Dissemination for Urban Vehicular Environments. IEEE Trans. Intell. Transp. Syst..

[25] Zhang R., Lu R., Cheng X., Wang N., Yang L. (2021). A UAV-Enabled Data Dissemination Protocol with Proactive Caching and File Sharing in V2X Networks. IEEE Trans. Commun..

[26] Almasoud A.M., Kamal A.E. (2019). Data Dissemination in IoT Using a Cognitive UAV. IEEE Trans. Cogn. Commun. Netw..

[27] Al-Omaisi H., Sundararajan E.A., Abdullah N.F. Towards VANET-NDN: A Framework for an Efficient Data Dissemination Design Scheme. Proceedings of the 2019 International Conference on Electrical Engineering and Informatics (ICEEI).

[28] Chowdhury D.R., Jain V.K., Maurya S. Travel Angle Based Fast Data Dissemination to Relevant Vehicles in VANET. Proceedings of the 2018 41st International Conference on Telecommunications and Signal Processing (TSP).

[29] Tei A.I., Doukha Z., Zafoune Y. Multi-criteria-based relay election for Data Dissemination in urban VANET. Proceedings of the 2019 International Conference on Theoretical and Applicative Aspects of Computer Science (ICTAACS).

[30] Hu C.-C. (2020). Peer-to-Peer Data Dissemination for Deadline-Sensitive Streaming in VANETs. IEEE Access.

[31] Vasudev H., Das D., Vasilakos A.V. (2020). Secure message propagation protocols for IoVs communication components. Comput. Electr. Eng..

[32] Shi Y., Eberhart R. A modified particle swarm optimizer. Proceedings of the 1998 IEEE International Conference on Evolutionary Computation Proceedings, IEEE World Congress on Computational Intelligence (Cat. No.98TH8360).

[33] Husain A., Singh S.P., Sharma S.C. (2020). PSO Optimized Geocast Routing in VANET. Wirel. Pers. Commun..

[34] Zhao C., Tang Y., Sun Q., Vasilakos A.V. (2019). Deep Direct Visual Odometry. IEEE Trans. Intell. Transp. Syst..

[35] Dya T., Blaise B.B., Betchewe G., Alidou M. (2021). Implementation of Particle Swarm Optimization Algorithm in Matlab Code for Hyperelastic Characterization. World J. Mech..

[36] Rathod S., Saha A., Sinha K. (2020). Particle Swarm Optimization and its applications in agricultural research. Food Sci. Rep..

[37] Gad A.G. (2022). Particle Swarm Optimization Algorithm and Its Applications: A Systematic Review. Arch. Comput. Methods Eng..

[38] Tripathi K.N., Yadav A.M., Sharma S.C. (2022). DDOS: Data dissemination with optimized and secured path using modified particle swarm optimization in vehicular communication network (VCN). Int. J. Inf. Technol..

[39] Bhardwaj A., El-Ocla H. (2020). Multipath Routing Protocol Using Genetic Algorithm in Mobile Ad Hoc Networks. IEEE Access.

[40] Elhoseny M., Shankar K. (2020). Energy Efficient Optimal Routing for Communication in VANETs via Clustering Model. Emerging Technologies for Connected Internet of Vehicles and Intelligent Transportation System Networks.

[41] Patel J., El-Ocla H. (2021). Energy Efficient Routing Protocol in Sensor Networks Using Genetic Algorithm. Sensors.

[42] Yang X.S., Watanabe O., Zeugmann T. (2009). Firefly Algorithms for Multimodal Optimization. Stochastic Algorithms: Foundations and Applications, SAGA 2009.

[43] Blum C. (2005). Ant colony optimization: Introduction and recent trends. Phys. Life Rev..

[44] Dahan F., El Hindi K., Ghoneim A., Alsalman H. (2021). An Enhanced Ant Colony Optimization Based Algorithm to Solve QoS-Aware Web Service Composition. IEEE Access.

